# Introduction to the Supplement ‘Coming together to fight cancer: a series of policy briefs taking stock of the implementation of Europe’s Beating Cancer Plan in Belgium’

**DOI:** 10.1186/s13690-024-01383-5

**Published:** 2024-09-26

**Authors:** Marie Delnord, Gabrielle Schittecatte, Jinane Ghattas, Marc Van Den Bulcke

**Affiliations:** 1 Sciensano, Belgian Institute of Health, 14 Rue Juliette Wyttsman, Brussels, 1050 Belgium; 2 Institut de recherche santé et société (IRSS), Université catholique de Louvain, Brussels, Belgium

**Keywords:** Cancer, Implementation research, Policy briefs, Cancer care and control, Co-design, Stakeholder engagement

## Abstract

Cancer is one of the main public health challenges globally. In Europe, it is also the second leading cause of mortality and incidence is likely to increase from nearly 4 million cases per year in 2020 to over 5 million new cases per year by 2040. Recognizing the urgency of tackling the entire disease pathway and supporting European Union (EU) Member States (MS), the European Commission (EC) launched two major initiatives: Europe's Beating Cancer Plan (EBCP) and the Mission on Cancer (MoC). Belgium is one of the few EU MS that has set up a dedicated structure, known as the Belgian EBCP Mirror Group (MG), to support the implementation of the EU strategy against cancer. The MG is a large national stakeholder platform coordinated by the Cancer Centre at Sciensano (Belgian Institute of Health). This Supplement between includes eight policy briefs developed by the MG on the most pressing needs to address in the Belgian cancer field. The policy briefs take into account relevant interventions at national level as well as Belgium's participation in European projects as of 2021-2023.

**Supplementary Information:**

The online version contains supplementary material available at 10.1186/s13690-024-01383-5.

## Background

Cancer is one of the main public health challenges globally. In Europe, cancer is also the second leading cause of mortality, and incidence is likely to increase from nearly 4 million cases per year in 2020 to over 5 million new cases per year by 2040 [1, 2]. Recognizing the urgency of tackling the entire disease pathway and supporting European Union (EU) Member States (MS), the European Commission (EC) has launched two major initiatives: Europe's Beating Cancer Plan (EBCP) and the Mission on Cancer (MoC). With a total of €4 billion earmarked, the EBCP is a policy-driven initiative addressing prevention, early detection, diagnosis and treatment, and an improved quality of life for cancer patients and survivors. The MoC leverages research and innovation to improve the lives of 3 million cancer patients by 2030. This Supplement is an opportunity to reflect on what has been achieved since the launch of the EBCP in the Belgian cancer field, and highlight where improvements are still needed.

## The Belgian EBCP Mirror Group

Cancer mortality rates in Belgium are among the lowest in the EU, and five-year cancer survival is above the average for most common cancers [3]. Still, there were 74.998 new diagnoses of cancer registered in 2021 (excluding non-melanoma skin cancer) [4], and 27 000 cancer-related deaths per year—representing a heavy burden for families and a significant loss for society at-large [5, 6]. In men, prostate cancer is the most common cause of new cancer diagnosis, followed by lung and colorectal cancer. In women, breast cancer is the most common followed by lung and colorectal cancer [4–6].

To support the implementation of the EU strategy against cancer (i.e. EBCP and MoC) in Belgium, the Minister of Social Affairs and Public Health, Frank Vandenbroucke, in consultation with the Federal Cabinet, the Federal Public Service Health, Food Chain Safety and Environment, and the National Health and Disability Insurance (NIHDI), mandated the Belgian Cancer Centre^1^ to coordinate this work. Belgium is one of the few EU Member States (MS) that has set up a dedicated structure, known as the Belgian EBCP Mirror Group (MG) to support the implementation of the EBCP and the Mission on Cancer.

To launch the MG, the Belgian Cancer Centre reached out initially to its collaborators and network for participation. Currently, over 400 representatives of federal/regional health agencies, universities, professional societies, patient/citizen organisations and industry participate on a volunteer basis to regular meetings and workshops.

**Fig. 1:**
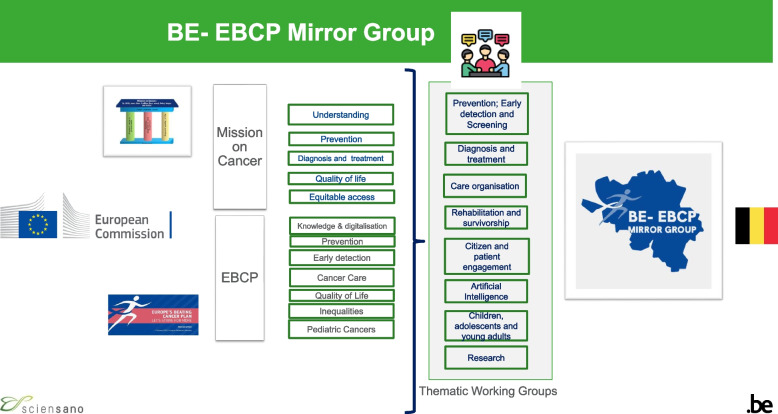
Organisation of the Belgian EBCP Mirror Group (MG) into Thematic Working Groups Note: At the launch of the MG, there were initially 8 TWGs. Since then, the MG structure evolved based on the input received from the field. TWG Research has matured into a separate entity the Belgian Cancer Research Alliance. As of August 2024 there are currently 6 TWGs. Please see the BE EBCP Mirror Group website for more information: https://www.beatingcancer.be/about/

As shown in Fig. 1, the MG which is a multi-stakeholder initiative is structured into Thematic Working Groups (TWGs). Each of the TWGs is chaired by a 'lead' from the Belgian Cancer Centre. The TWGs are open, meaning that any interested individual or expert working in the cancer field can sign up to TWGs via the project webpage: www.beatingcancer.be. Participants can sign up to as many TWGs as they wish based on their interests and professional experience. TWG Members receive updates on running EU calls and projects from the Cancer Centre, which is the acting secretariat of the MG. The MG provides a forum to discuss where EC funding is most needed for cancer patients and in the health system. TWG members contribute to consultations and to the development of recommendations for decision- and policy-makers. This participatory approach aims to sustain a high level of engagement of the field with the EBCP aims and objectives; thereby strengthening the impact of the EBCP and MoC in Belgium. It also allows to better streamline cancer-related communication and activities at the national and regional level.

## Implementation of the EBCP and Mission on Cancer in Belgium 2021–2023

Since the launch of the EBCP and the Mission on Cancer in 2021, there have been over 80 EC calls^2^ published in the areas of cancer prevention, diagnosis and treatment, care organisation and survivorship. The Cancer Centre, as acting secretariat of the MG, monitors Belgium's participation in EU cancer projects whether these are funded through EU4Health, Horizon Europe or other EC Work programmes. Call participation of EU-MS is reported in the Horizon Europe dashboard, and from other EC webpages for different work programmes. Whereas all EU funded projects are listed in ‘The Community Research and Development Information Service’ (CORDIS) website, these are not tagged under the keyword “EBCP”. In general, information on EBCP and MoC implementation (i.e. on EU-MS participation in EU cancer projects) is somewhat fragmented, which warrants the support of the MG in compiling this overview for Belgium. Moreover, some information on the outcomes of competitive calls and which institutions applied are considered confidential, with access granted only to EU-MS nominated National Contact Points (NCPs).

How EC funding is used in the health system and ‘who does what’ is an issue of public accountability and transparency. This is particularly relevant for the EBCP and MoC outputs that should be easily findable and accessible to those who need it for intervention. Taking stock of what has been implemented shows where strategic investments are made.

**Fig. 2:**
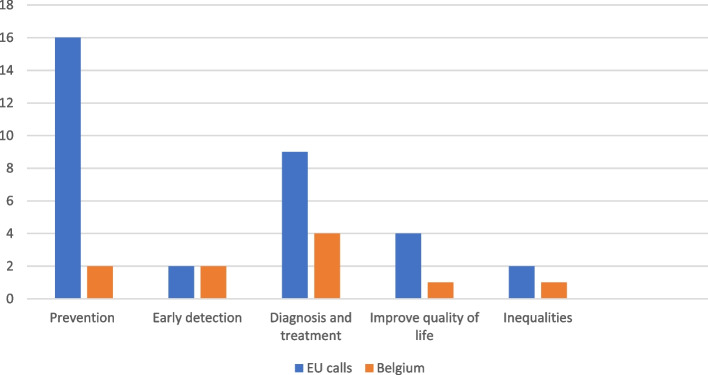
Belgium’s participation in the EBCP projects for the period 2021–2023* *Note: [1] The EU calls listed here are those related to cancer in the EU4Health programme. We are using the participation of the Cancer Centre in the EU4Health Programme as a proxy to Belgium’s participation in the EBCP; [2] Some calls can fund several projects. When this is the case, the projects are counted just once for a better correspondence with the number of EU call openings

**Fig. 3:**
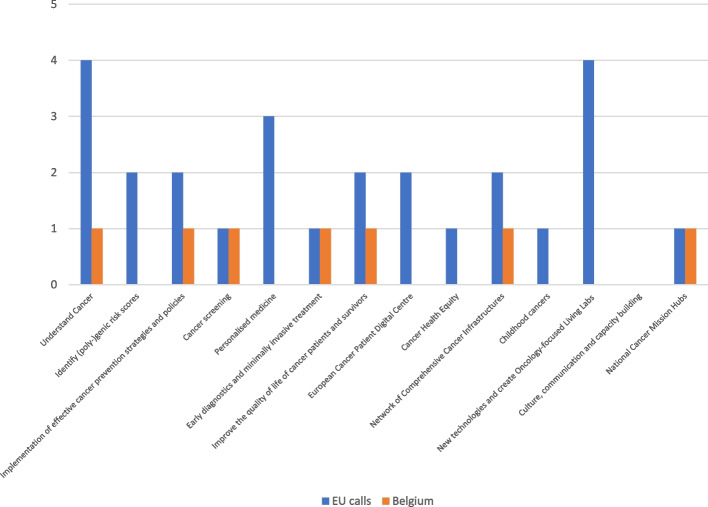
Belgium’s participation in the Mission on Cancer for the period 2021–2022 Note: Latest available data on EU calls and BE projects funded by the Horizon Europe programme Cluster 1 Health and the Mission on Cancer. The calls are categorized based on their link to the 13 Mission on Cancer recommendations; [2] Some calls can fund several projects. When this is the case, the projects are counted just once for a better correspondence with the number of EU call openings

As shown in Figs. 2 and 3, Belgium participates in European projects across all cancer domains, casting a wide net for improvements in cancer prevention, diagnosis and treatment, care and survivorship. More information on the EU projects of the Cancer Center for the period 2021–2023 is available in Additional file 1.

Although participation seems lower in the area of prevention in comparison to the number of EU calls that were open, this is not necessarily a reflection of the number, nor the quality or performance of existing cancer prevention activities in Belgium. For instance, the number of European projects and calls shown in Figs. 2 and 3 does not reflect the amount of funding allocated overall, or to the Belgian partners. In any given call topic/area, low EU-MS participation can be due to several factors. An important obstacle to EBCP implementation and participation in EU projects can be, for example, the lack of alignment of certain EU calls with existing national programs and interventions. MS participation is also rationalized based on contextual factors, such as governmental priorities given the policy cycle, existing infrastructures, the availability of co-funding, human resources, as well as cultural norms and values.

## Identifying cancer gaps and needs in Belgium

The Belgian EBCP Mirror Group, was consulted to develop the 8 policy briefs included in this Supplement. Policy briefs are a well know knowledge transfer tool to provide a concise summary of evidence, suggest policy options, or advocate for particular courses of action [7, 8]. The policy briefs were formulated iteratively and are the result of a standardized exercise. The TWG participants were asked to: 1) Identify the main issues, needs and gaps for Belgium in key cancer areas; 2) Provide a state-of-play of relevant EU and Belgian projects and interventions addressing the issue(s) and 3) Outline policy and practice recommendations to improve cancer outcomes in Belgium given the gaps identified and ongoing projects. Table 1 below summarizes the topics and issues that are covered in this Supplement. More information is available in the corresponding policy briefs.

**Table 1:** Cancer policy and practice needs in Belgium as informed by the Thematic Working Groups of the Belgian EBCP Mirror Group (MG)

**TWG Area/ policy brief topic**	**Problem statement for Belgium **
Prevention & Early detection and screening	Lack of comprehensive registration and data linkage for cancer mortality, incidence, screening and vaccination (HPV/HB) in Belgium
	New innovations in screening that need to be leveraged for both new screening programs (prostate and lung), as well as whole population screening
Diagnosis & Treatment	Lack of adequate funding and structural organization to facilitate access to precision (hemato-)oncology
	Lack of reporting platforms to link real world data and treatment and outcomes
	Difficulties finding independent funding for high-quality academic clinical trials in Belgium
	Lack of organisational support to access innovative academically-developed cell therapy in a timely, safe and affordable way
	A structured approach to coverage of evidence development for assessing and implementing emerging radiotherapy innovation is lacking at the national level
Artificial Intelligence	Need for more transparency in AI algorithms, adding to ethical issues on the use of sensitive data; need for testing of new AI tools
	Gap in development of AI for clinical decision support tools and integration with external knowledge base
Care Organisation	Need for better integration and concentration of care; comprehensive cancer centers and networks are not formally recognized
	Despite advances in innovative care, including in screening, diagnosis, and treatment, there is a lack of legal framework organising these initiatives
Children and Adolescent and Young Adults (CAYAs)	Special needs to be addressed in a patient- and cancer-driven way
	Gap in adoption of innovations into care for paediatric and AYA with cancer (molecular profiling, clinical trials)
	Need to support and expand pilot projects aimed at identifying genetic predisposition to prevent and detect early cancer
	Lack of support for structuredmulti-disciplinary long-term follow-up as well as transition path between paediatric and adult haemato-oncology
Survivorship	Amount of cancer survivors are increasing, but survivorship is often an afterthought in care organisation
	Missed opportunities to integrate survivorship care in standardized care pathways
	No requirements (long-term) on survivorship care in the existing legal basis of Oncological Care Programmes, and lack of quality indicators monitoring cancer survivorship in quality assurance schemes (such as certification of centres)
	No information system in Belgium for survivorship, including no electronic records available to collect physical or psychosocial information from survivors
Research	Challenges translating outcomes from research : “Bench-to-Bed and Bed-to-Bench”
	Gaps in the collaboration and fragmentation between actors in the research field, hampering the development of fundamental and translational research
	Certain disciplines (e.g., psycho-social sciences, behavioural sciences ) are not consistently included in research projects or EU calls
Patient & Citizen Engagement	Lack of systematic and organized engagement of patients, including in health data
	Results from citizen engagement initiatives, even when they produce clear policy outputs, are rarely really taken into account when new policies are designed
	Need for sustainable support (financial and technical) for patients to be engaged continuously

## Strengths and limitations

This supplement provides a comprehensive gaps and needs analysis of key cancer topics in Belgium in the light of the European strategy against cancer (i.e. EBCP and Mission on Cancer). In particular, the participatory approach used to develop the Supplement allowed members of the MG to agree on baseline problem definition, and identify ways to overcome remaining challenges in cancer care and control. Nevertheless, this supplement is not a formal evaluation of cancer performance in Belgium. The policy briefs are limited to the collective knowledge and opinions of the by-lined authors and TWGs. There may also be power imbalances that favour the input and positions of those with the time and resources to participate in the TWGs [9]. Smaller organisations for instance, may have been underrepresented in the meetings leading up to the policy briefs.

## Conclusion

Cancer inequalities in Europe are related to differences in population characteristics, national or regional policies, and access to high quality diagnostics and care. Participating in EU projects can help reduce those inequalities by facilitating the uptake of innovation and the exchange of best practices across MS. Efficient interventions can be introduced or scaled-up when key actors in policy and practice work closely together [10, 11]. This supplement identifies where concerted efforts are needed, to  pave the way forward for improvements across the cancer disease continuum in Belgium.

## Supplementary Information

Supplementary Material 1.

## Data Availability

All data generated or analysed during this study are included in this published article [and its supplementary information files].

## Abbreviations

BE: Belgian/Belgium
EBCP: Europe’s Beating Cancer Plan
EC: European Commission
EU: European Union
MoC: Mission on Cancer
MS: Member State
NIDHI: National Health and Disability Insurance
TWG: Thematic Working Group

## Footnotes

1 The Belgian Cancer Center is hosted at Sciensano, the Belgian Institute of Health.

2 the EU4Health Work Programme and Horizon Europe fund the EBCP and the Mission on Cancer respectively. Other funding programmes can also be relevant for cancer such as Digital Europe.
