# Supplementary material for: Introduction to the Supplement ‘Coming together to fight cancer: a series of policy briefs taking stock of the implementation of Europe’s Beating Cancer Plan in Belgium’
Source: Arch Public Health. 2024 Sep 26;82(Suppl 1):164. doi: 10.1186/s13690-024-01383-5 (PMC11426068; doi:10.1186/s13690-024-01383-5)
Supplement: Supplementary file 1 — Supplementary Material 1. [file 13690_2024_1383_MOESM1_ESM.docx]

Additional File 1. List of EU projects linked to Europea’s Beating Cancer Plan (EBCP) and the Mission on Cancer (MoC) in which the Belgian Cancer Center participates for the period Feb 2021-June 2023*

| **Theme** | **EC PROJECTS** | **Expected outcomes** | **PROGRAM** | **START DATE** | **Time (Months)** | **Belgian interest/impact and partners** |
| --- | --- | --- | --- | --- | --- | --- |
| Early detection | **Canscreen-ECIS** | Indicators Cancer screening programs | EU4Health | 01/08/2022 | 18 | Update indicators for monitoring cancer screening programs; integration in Belgian screening programs assessments  Participation Belgium: Sciensano (SC), CVKO |
| Quality of life | **eCAN** | Policy on telemonitoring & teleconsultation | EU4Health | 15/09/2022 | 24 | Develop framework for introduction of safe, secure and affordable telemedicine and telemonitoring approaches; integrate in digital health tools in Belgian HCS Participation Belgium: SC, FOD-VVVL, RIZIV, UZA |
| Diagnosis & treatments | **CAN.HEAL** | Proposals for implementation for new D&T/PHG in cancer | EU4Health | 01/11/2022 | 24 | Develop recommendations for: standardized multi-centric MTBs and tools, clinical utility of use of complex Genome profiling testing in medical and population-based applications, validation and assessment protocols for liquid biopsy testing; tools for education and training of professionals on genomics; assessment framework for innovative approaches for HCS; develop good/best practices for new diagnostics/interventions/treatments in Personalized Medicine and Health; citizen ELSI considerations on primary use of medical data  Participation Belgium: SC, Ugent, IJBordet, KULeuven, ULiège, Jessa Hospital |
| Care organisation | **CraNE** | Comprehensive Cancer Care Centers and CCC networks | EU4Health | 01/10/2022 | 24 | Develop a Belgian vision on the concepts of CCC centers and CCCN; implications for Belgian hospitals/institutes to participate in EC initiatives; national AYA/cancer ERNs/Pancreas/Oesopagus networks; initiate thinking on establishing CCC research infrastructures  Participation Belgium: SC, UZA |
| Diagnosis & treatments | **JANE** | 7 New Networks of Expertise (Survivorship, palliative care, AYA, Cancer with poor prognosis, omics, complex treatments and infrastructures, precision preventive health) | EU4Health | 01/10/2022 | 24 | Identify interests and benefits for Belgian professionals; liaise with and integrate in the new NoE  Participation Belgium: SC, Ugent, IJBordet |
| Prevention | **PERCH** | Vaccination campaigns and monitoring | EU4Health | 01/10/2022 | 30 | Support HPV vaccination campaigns for increasing coverage; integrate the developed linked registration tool for vaccination and screening  Participation Belgium: SC |
| Diagnosis & treatments | **JA ORION** | Framework for monitoring EBCP implementation | EU4Health | Accepted in July 2023 | 24 | Develop a framework and tool to optimally support the implementation of the EBCP at Belgian level  Participation Belgium:SC |
| Prevention | **JA –Prevent NCDs** | Consortium on Cancer Prevention | EU4Health | Jan-24 | 48 | Recommendations for establishing the “Consortium on Cancer Prevention” at EU level – implementation of interventions for prevention of cancer and NCDs |
| Prevention | **AG EHDS pilot** | Pilots for EHDS | EU4Health | early 2023 | 36 | Development of a usecase on cancer allowing exchange of data for linking population data with Cancer registry to identify people at risk in health populations; importance for underpinning several health questions with data coming from several EU member states - NGS diagnostics, Return to work, cancer screening/vaccination initiatives in Belgium Participation Belgium:: SC, |
| Diagnostics and treatment | **AG EUCAIM** | Cancer Images infrastructure | DIGITAL  EUROPE | 01/11/2022 | 48 | Develop platform for Cancer Images data for research and medical use; link with Belgian EHDS initiative and AI tools development Participation Belgium:: SC, GZA |
| Diagnosis & treatments | **GDI** | Genomics data infrastructure | DIGITAL  EUROPE | 01/11/2022 | 48 | Develop platform for Genomics sequencing data for research and medical use; link with Belgian EHDS initiative, the Belgian Human Genome initiative and AI tools development; link with NGS diagnostics in HCS  Participation Belgium: SC, VIB, Imec |
| Research | **UNCAN.eu** | Roadmap research in cancer | HORIZON  EUROPE | 01/09/2022 | 15 | Develop the cancer research roadmap for the EU to better understand cancer by 2030; impact on Belgian cancer research, opportunities for participation in high-impact international initiatives Participation Belgium: ULB, SC |
| Care organisation | **CSA_CCI4EU** | Comprehensive Cancer Infrastructures for research | HORIZON EUROPE | May 2023 | 36 | Develop the Belgian network of Comprehensive Cancer Care and Research Infrastructure in order to guarantee optimal participation in future EU4Health/MoC initiatives |
| Research/Other | **CSA-ECHOs** | National Mirror groups for MoC implementation | HORIZON EUROPE | May 2023 | 36 | Be EBCP Mirror group acts as a benchmark for the EU network of Cancer Mission Research Hubs |

Note. (1) We have listed above all projects that underpin the EBCP and Mission on Cancer objectives that were accepted/launched after the start of the EBCP in February 2021 until June 2023. These projects are funded by the EU4Health, Horizon Europe or Digital Europe programmes; (2) Projects are sorted by start date; (3) Results from the 2023 Cancer Mission calls are not available yet, and could not be included; (4) The Belgian Cancer Center participates in other EU projects which are not listed here due to starting prior to the EBCP. The full list of projects in which the Belgian Cancer Center participates is publicly available from their website: https://www.e-cancer.be/fr/le-centre-du-cancer
